# Trends in the Use of Promotional Language (Hype) in Abstracts of Successful National Institutes of Health Grant Applications, 1985-2020

**DOI:** 10.1001/jamanetworkopen.2022.28676

**Published:** 2022-08-25

**Authors:** Neil Millar, Bojan Batalo, Brian Budgell

**Affiliations:** 1 Department of Computer Science, University of Tsukuba, Tsukuba, Japan; 2 Division of Life Sciences, Canadian Memorial Chiropractic College, Toronto, Ontario, Canada

## Abstract

**Importance:**

The integrity of the grant application process is important to the success of the entire research enterprise. However, little information is available concerning the prevalence and evolution of subjective or promotional language (“hype”) that has the potential to undermine objectivity in the writing and evaluation of grant applications.

**Objective:**

To assess changes over time in the use of hype in abstracts of National Institutes of Health (NIH) grant applications.

**Design, Setting, and Participants:**

This cross-sectional study assessed the prevalence of promotional adjectives in abstracts in the NIH archive from 1985 to 2020.

**Main Outcomes and Measures:**

From all abstracts in the NIH RePORTER (Research Portfolio Online Reporting Tools: Expenditures and Results) archive, adjectives were automatically extracted, and their frequencies in the most recent year (2020) were assessed relative to the start year (1985). Adjectives that shifted significantly in frequency and that carried a promotional sense (ie, hype) were retained, and patterns of change were assessed by plotting yearly frequencies (1985-2020). By grouping the adjectives based on shared semantic properties, broad meanings commonly expressed by hype were identified. Absolute change was measured as the difference in normalized frequency between 1985 and 2020. Relative change was measured as the percentage change in normalized frequency in 2020 relative to 1985, or the first year of occurrence.

**Results:**

In total, 901 717 abstracts were analyzed and 139 adjective forms were identified as hype. Among these 139 adjective forms, 130 hype adjectives increased in frequency by 7690 words per million (wpm) (mean [SD] relative increase, 1378% [3132%]), while 9 hype adjectives decreased in frequency by 686 wpm (mean [SD] relative decrease, 44% [18%]). The largest absolute increases were for the terms *novel* (1054 wpm), *critical* (555 wpm), and *key* (461 wpm), while the largest relative increases were for the terms *sustainable* (25 157%), *actionable* (16 114%), and *scalable* (13 029%)*.* Hype most often serves to promote the significance, novelty, scale, and rigor of a project; the utility of the expected outcomes; the qualities of the investigators and research environment; and the gravity of the problem; as well as conveying the personal attitudes of the applicants.

**Conclusions and Relevance:**

Levels of hype in successful NIH grant applications have increased over time from 1985 to 2020. The findings in this study should serve to sensitize applicants, reviewers, and funding agencies to the increasing prevalence of subjective, promotional language in funding applications.

## Key Points

**Question:**

Has the use of “hype” (promotional language) in the abstracts of successful National Institutes of Health applications increased since 1985?

**Findings:**

This cross-sectional study of 901 717 National Institutes of Health abstracts from 1985 to 2020 shows that applicants described their work in increasingly subjective terms and relied on promotional language and appeals to emotion (ie, 130 adjective forms identified as hype increased in frequency).

**Meaning:**

This study suggests that applicants, reviewers, and funding agencies should be aware of the increasing prevalence of promotional language in funding applications.

## Teaser

This cross-sectional study assesses changes from 1985 to 2020 in the use of subjective, promotional language in abstracts of National Institutes of Health (NIH) grant applications.

## Introduction

The evolution of language, including the language of biomedicine and health, is a natural phenomenon and, in and of itself, is neither good nor bad. However, studies have identified what may be a trend in writing strategies (so-called spin) that has the potential to undermine the fidelity of scientific reporting.^1^ Although the definition may vary from study to study, spin has been characterized as “a specific intentional or unintentional reporting that fails to faithfully reflect the nature and range of findings and that could affect the impression the results produce in readers.”^2^^(p2613)^ Therefore, spin encompasses such practices as selective reporting of data, a lack of focus on adverse effects, and claiming or implying treatment effects that are not justified by statistical analyses of the data.

Although methods differ, studies of spin demonstrate that it is not uncommon in one genre of medical literature: the peer-reviewed research article.^3,4,5,6^ Setting aside for the moment the question of whether spin in medical articles results in harm to patients, the identification and modulation of spin in the peer review process appear to be problematic.^7,8^ In part, the challenge for authors, reviewers, and readers may be a paucity of information on how spin is realized at the sentence level. Thus, we are advised to look out for spin, but not told precisely what the language of spin looks like.

Researchers in linguistics, however, have drawn attention to one quite specific mechanism to implement spin, and this mechanism is termed *hype*. Hype has been defined as hyperbolic and/or subjective language that may be used to glamorize, promote, or exaggerate aspects of research.^9^ Hype, therefore, includes rhetorical devices that are subsumed under the broad label of spin, which is focused on the misreporting and misrepresenting of findings. Although perhaps a more subtle mechanism, hype can nonetheless be characterized by a particular lexicon, which may render identification more reliable and quantifiable.

As examples of the increase of hype in biomedical writings, it has been demonstrated that the relative prevalence of value-laden adjectives, including *important*, *critical*, and *original*, increased in the clinical literature from 1985 to 2005.^10^ In addition, a study of PubMed abstracts from 1974 to 2014 revealed an almost 9-fold increase in the prevalence of “positive” words (eg, *robust*, *novel*, *innovative*, and *unprecedented*) vs an approximately 2.6-fold increase in “negative” words (eg, *disappointing*, *inadequate*, *ineffective*, and *insignificant*).^11^ Concern has been expressed that “overestimation of research findings directly impairs the ability of science to find true effects and leads to an unnecessary focus on research marketability.”^11^^(p3)^ Even in a broad range of academic literature from 1965 to 2015, substantial increases have been demonstrated in writing strategies that could be used to impose judgments on the reader or instruct the reader on how they should interpret the authors’ writings.^12^ In a separate study during the same period, the authors also noted what they called a massive increase in the choice of words used to “promote, embellish, or exaggerate” various aspects of research reports.^13^ Thus, more precise information is emerging regarding how authors use hype to achieve spin in research reports.

Whether similar trends are present in another genre, the grant application, has yet to be explored on a large scale. Given the importance that the grant application holds in the larger research ecosystem, there should be concern that an inappropriate degree of salesmanship vs scientific merit in grant applications could undermine the integrity of research funding mechanisms. The purpose of the present study was to analyze the changes in word choices in abstracts of successful National Institutes of Health (NIH) grant applications from 1985 to 2020, with particular attention to the use of judgment-laden words (ie, hype).

## Methods

### Study Design

We analyzed all abstracts contained within the NIH archive of funded projects. We restricted our analysis to adjectives because they are the word class most associated with evaluation^14^ and can be reliably identified automatically.^15^ By comparing abstracts published in 2020 (the most recent year in the archive) with those published in 1985 (the earliest year), we generated a list of adjectives showing statistically significant changes in frequency. From this list, we manually identified adjectives that functioned as hype and then categorized them based on shared semantic properties. We assessed the overall patterns of change in the data for all years (1985-2020), thereby also identifying hyping adjectives that only appeared recently but that have undergone a significant increase in prevalence. Because the study did not involve human participants, approval was waived by the University of Tsukuba institutional review board. This study was designed to be compliant with the relevant items of the Strengthening the Reporting of Observational Studies in Epidemiology (STROBE) reporting guideline checklist for cross-sectional studies.

### Statistical Analysis

#### Data Preprocessing

From the NIH ExPORTER (Exported Research Portfolio Online Reporting Tools: Expenditures and Results) system,^16^ we downloaded all records (approximately 2.17 million) linked to projects funded by the NIH and related agencies from 1985 to 2020. We removed duplicates and records with missing abstracts and automatically annotated the abstracts for grammatical parts of speech using the Stanza NLP package (Stanford NLP Group).^17^ We then loaded the resulting text corpus, along with metadata for year of first occurrence, into the CQPweb corpus analysis system (Lancaster University).^18^

#### Assessing Changes in Adjective Frequency

We used keyword analysis—a corpus linguistic method in which word frequency lists from 2 corpora are compared to generate a list of words, referred to as *keywords,* that occur statistically significantly more often in one corpus than the other.^19^ In plain language, we compared the frequencies of adjectives in abstracts from 2020 with those in abstracts from 1985. As is standard, we used the log-likelihood test to compare frequencies and applied the Bonferroni correction to maintain a studywide α of .05. All statistical tests were 2-sided, and analyses were performed in the R statistical programming environment, version 4.1.0 (R Group for Statistical Computing).^20^

#### Identifying Hype Adjectives

Two of us (a linguist [N.M.] and a biomedical researcher [B. Budgell]) first independently coded the nonhype adjectives (eg, technical adjectives and proper adjectives), which were then removed. The researchers then independently coded the remaining candidate adjectives as hype if more than 30% of the usage was judged to be promotional. This coding involved reading concordance lines (ie, sentences from the corpus of abstracts) showing how the adjective was used in context in 2020. For adjectives with fewer than 100 occurrences, all instances were read. For adjectives with more than 100 occurrences, a sample of one-third of the occurrences or at least 500 occurrences were read. Adjectives were identified as hype by judging whether or not they could be removed or replaced with a more objective or neutral alternative word without altering the information within the sentences—examples are shown in the following 3 extracts, with hype adjectives italicized: (1) “As such, development of *novel* radiosensitizing agents is of *crucial* importance…”; (2) “We will use a *powerful* new methodology, tandem mass spectroscopy, for *accurate* detection of natural ceramide species…”; and (3) “InDevR has an *excellent* track record in translating *innovative* concepts into *impactful* products.” In both stages, the researchers achieved strong interrater agreement (nonhype adjectives, κ = 0.80; hype adjectives, κ = 0.82) before resolving disagreements by discussion.

#### Identifying Hype Categories

We grouped the hype adjectives based on shared semantic properties to capture the broad meanings that applicants use hype to express. This process was aided by using WordNet (Princeton University), a word sense disambiguation tool.^21^ It was an iterative and primarily inductive process conducted by the first author (N.M.), followed by discussion with the other authors (B. Batalo and B. Budgell) and modification of categories. In cases in which an adjective could belong to more than 1 group, it was assigned according to the more prevalent meaning.

#### Outcome Measures

To enable comparison, we normalized frequency counts for each year to a common base—words per million (wpm). We used 2 outcome measures: (1) absolute change, which is the difference in normalized frequency between 1985 and 2020, and (2) relative change, which is the percentage change in normalized frequency in 2020 relative to 1985, or the first year of occurrence—not all current hype adjectives existed in the literature in 1985.

## Results

The corpus of NIH abstracts contained 901 717 abstracts (355.8 million words) and approximately 36.4 million adjectives. There was no statistically significant difference in the overall prevalence of adjectives between 1985 and 2020 (103 965 wpm vs 104 045 wpm). Comparing these 2 years, 1888 adjective forms showed a statistically significant change in frequency (*P* < .05). Of these, 139 adjectives were judged to function as hype (eTable in the Supplement).

The pattern was one of growth in the use of hype. Of the 139 hype adjectives, 130 increased in frequency by 7690 wpm, with a mean (SD) relative increase of 1378% (3132%). In contrast, only 9 hype adjectives decreased in frequency by 686 wpm and a mean (SD) relative decrease of 44% (18%). In 1985, 72% of NIH abstracts contained 1 or more of these hype adjectives. By 2020, this percentage increased to 97% of abstracts (*P* < .001), with the interim years, on the whole, showing year-on-year increases.

**Figure 1.**
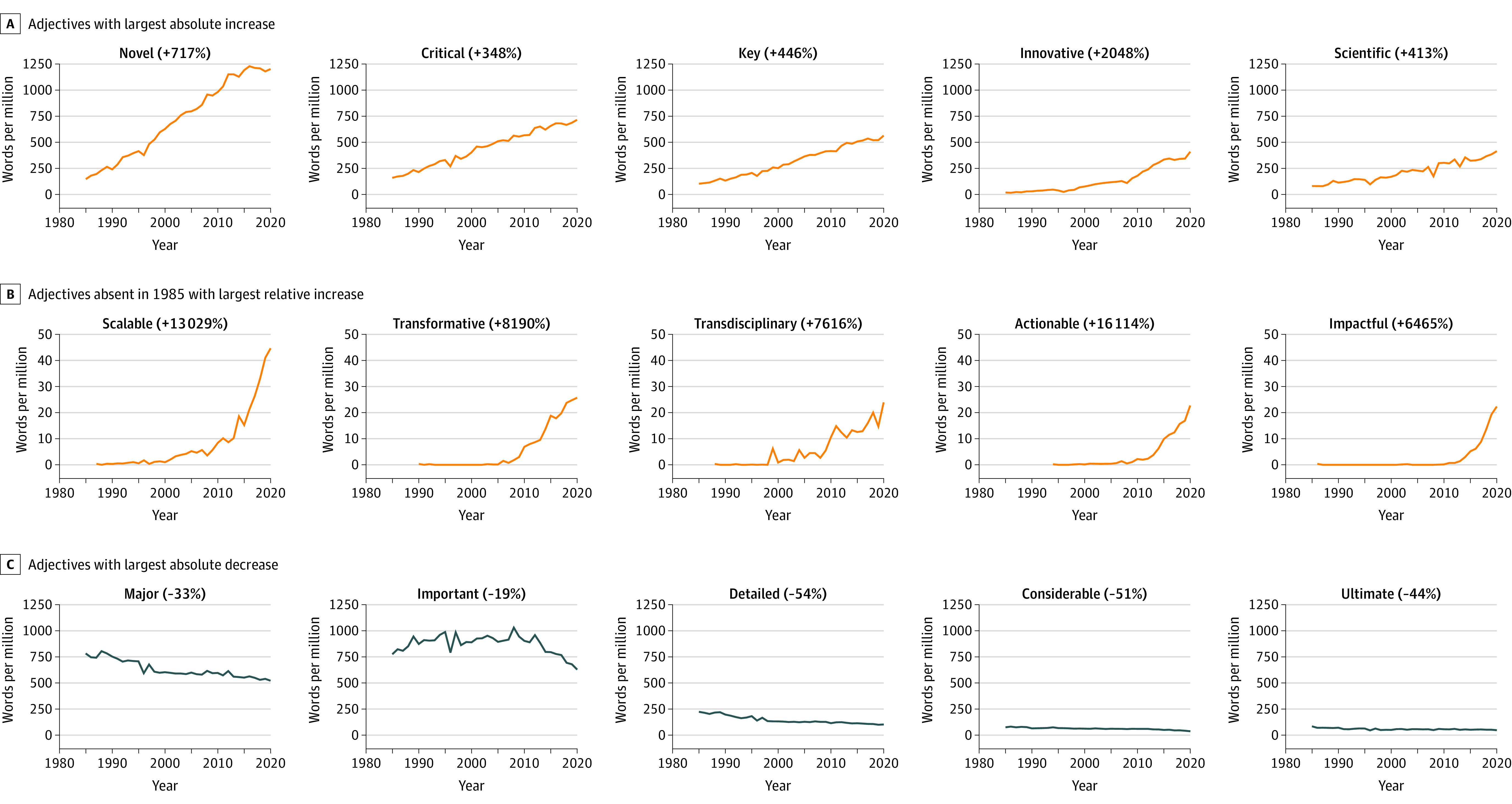
Yearly Frequencies for Selected Hype Adjectives From 1985 to 2020 in Words per Million Percentages indicate the overall relative change from 1985 to 2020.

The plots in Figure 1 show the frequency per year (1985-2020) for selected hype adjectives (see eFigure in the Supplement for all plots). Figure 1A shows adjectives with the largest absolute increases—*novel* (1054 wpm), *critical* (555 wpm), *key* (461 wpm), *innovative* (391 wpm), and *scientific* (334 wpm). A total of 19 adjectives were absent from the NIH abstracts in 1985 but have since gained popularity; Figure 1B shows those with largest relative increases—*actionable* (16 114%), *transdisciplinary* (7616%), *scalable* (13 029%), *transformative* (8190%), and *impactful* (6465%). The adjective *sustainable*, which occurred only once in 1985, increased by 25 157%. Other adjectives were absent from the NIH abstracts in 1985 but have since gained popularity, and they include *renowned*, *incredible*, *groundbreaking*, and *stellar*. The largest absolute decreases were for *major* (−261 wpm), *important* (−147 wpm), *detailed* (−121 wpm), *considerable* (−38 wpm), and *ultimate* (−37 wpm) (Figure 1C).

**Figure 2.**
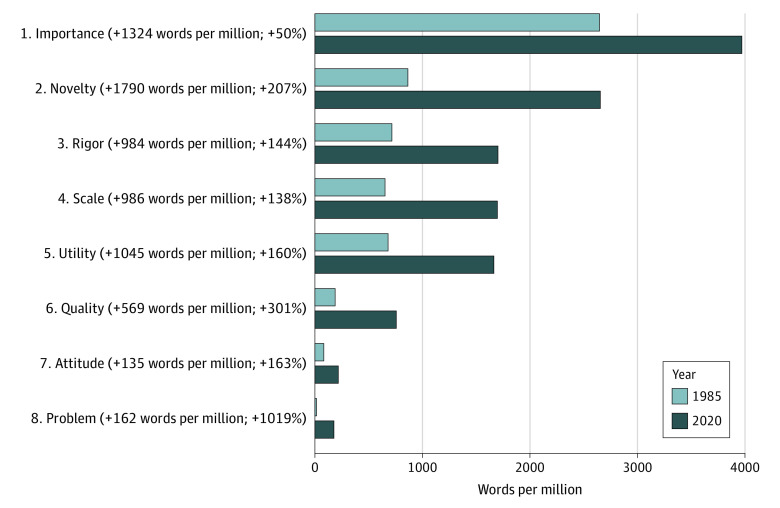
Frequency of Broad Semantic Categories in 1985 and 2020 in Words per Million Percentages indicate the overall relative change from 1985 to 2020.

The hype adjectives occupied 8 broad semantic categories (importance, novelty, rigor, scale, utility, quality, attitude, and problem), which are shown in the Box. The Table gives examples of hype adjectives in context, and Figure 2 shows their overall frequency shifts.

Box. **Broad Semantic Categories and Hype Adjectives**

1. *Importance:* compelling, critical, crucial, essential, foundational, fundamental, imperative, important, indispensable, invaluable, key, major, paramount, pivotal, significant, strategic, timely, ultimate, urgent, and vital;
2. *Novelty:* creative, emerging, first, groundbreaking, innovative, latest, novel, revolutionary, unique, unparalleled, and unprecedented;
3. *Rigor:* accurate, advanced, careful, cohesive, detailed, nuanced, powerful, quality, reproducible, rigorous, robust, scientific, sophisticated, strong, and systematic;
4. *Scale:* ample, biggest, broad, comprehensive, considerable, deeper, diverse, enormous, expansive, extensive, fastest, greatest, huge, immediate, immense, interdisciplinary, international, interprofessional, largest, massive, multidisciplinary, myriad, overwhelming, substantial, top, transdisciplinary, tremendous, and vast;
5. *Utility:* accessible, actionable, deployable, durable, easy, effective, efficacious, efficient, generalizable, ideal, impactful, intuitive, meaningful, productive, ready, relevant, rich, safer, scalable, seamless, sustainable, synergistic, tailored, tangible, transformative, and user-friendly;
6. *Quality:* ambitious, collegial, dedicated, exceptional, experienced, intellectual, longstanding, motivated, premier, prestigious, promising, qualified, renowned, senior, skilled, stellar, successful, talented, and vibrant;
7. *Attitude:* attractive, confident, exciting, incredible, interesting, intriguing, notable, outstanding, remarkable, and surprising; and
8. *Problem:* alarming, daunting, desperate, devastating, dire, dismal, elusive, stark, unanswered, and unmet.

**Table.** Sample Sentences Showing Examples of Hype Adjectives in Context^a^

Category and examples	NIH grant
**1. Importance**	
“Further, a unique and *key* aspect of this program is the sharing of common mouse strains, reagents…”	R01AG032179
“There remains an *imperative* need for more advanced PACT breast imaging technologies.”	R35CA220436
“Addressing this severe knowledge gap in one of the most *fundamental* aspects of cytoskeletal biology is *paramount* to understanding how actin functions in cells.”	R35GM137959
**2. Novelty**	
“The proposed methods offer a *revolutionary* innovation and will be a game-changer in the…”	R43EB027535
“These *innovative* and *novel* studies will provide essential new information about the regulation of…”	R01HL084494
“We propose to go deep in analyzing a very *uniqu***e** and *unprecedented* large scale human genomic data set for aging research.”	R01AG055501
**3. Rigor**	
“Associates will conduct *rigorous* experiments that test hypotheses related to the effects of…”	R44CA073348
“Our emphasis is on *scientific* rigor and communication using the latest tools available with award-winning and collaborative dual mentoring…”	T32GM136615
“The GCRC provides specialized nursing care with *sophisticated* and *careful* monitoring of…”	M01RR000051
**4. Scale**	
“The *vast* scale of this screen sets this project apart from many previous investigations…”	R01GM124365
“The magnitude of the need for a tool for systemic delivery of peptides is *huge* so as to create an *enormous* market potential for cancer therapeutics.”	R43CA094699
“This dynamic approach resulted in the development of *myriad* infrastructure, programs, and services…”	UL1RR024153
**5. Utility**	
“The overall goal of this project is to employ rigorous empirical methods to develop and test care innovations that expand the scope of HIV care in a *sustainable*, *scalable*, and *impactful* way.”	UL1RR024153
“…will yield an *easy*-to-use benchtop instrument capable of rapid and accurate size measurements…”	R44TR003256
“…programs resulting from the *synergistic* partnership include an innovative pilot project…”	UL1RR025008
**6. Quality**	
“This includes having a *qualified* director and *skilled* staffs to actively consult on experimental design…”	ZICAG000618
“…along with expert consultants, who possess *stellar* track records of training junior scientists…”	K01MH119216
“The OCTSI will fundamentally change biomedical research to create a *vibrant* academic home for clinical/translational investigation.”	UL1RR024140
**7. Attitude**	
“For these aims, the chick embryo provides an *excellent* model for embryonic development with *incredible* accessibility for observation and manipulation, without the confounds of maternal behavior.”	F31NS118867
“The current funding cycle has revealed *remarkable* developmental processes that occur during…”	P01AI083211
“This proposal focuses on several questions related to these *intriguing* preliminary findings.”	R01HL057502
**8. Problem**	
“Thus, there is a *dire* need for effective interventions that can promote weight loss maintenance.”	I01HX000690
“…an urgent *unmet* need exists to identify potential targets to develop such therapies.”	R21AR065638
“Despite extensive studies, the molecular mechanism of ABC transporters remains *elusive* and many questions remain *unanswered*…”	R01GM076440

Abbreviations: ABC, ATP-binding cassette; GCRC, General Clinical Research Center; NIH, National Institutes of Health; OCTSI, Oregon Clinical and Translational Science Institute; PACT, photoacoustic computed tomography.

^a^ Hype adjectives are italicized.

Adjectives in group 1, “importance,” are associated with claims of significance, priority, and urgency (eg, *key*, *paramount*, and *imperative*) and usually serve to promote the necessity for the research. Group 2, “novelty,” comprises adjectives that, on the whole, emphasize originality and newness (eg, *innovative*, *unique*, and *unprecedented*). Group 3, “rigor,” relates to claims of diligence and methodological quality (eg, *careful*, *rigorous*, and *scientific*). Adjectives in group 4, “scale,” are associated with positive expressions of magnitude, breadth, depth, and rank (*enormous*, *myriad*, and *vast*). Adjectives in group 5, “utility,” promote efficacy, usefulness, and practicality (eg, *effective*, *impactful*, and *easy*), usually regarding the expected outcomes or choice of method. Group 6, “quality,” comprises adjectives associated with characteristics, qualities, and credentials of individuals (usually the researchers [eg, *qualified*, *skilled*, and *talented*]) or the research environment (eg, *premier*, *renowned*, and *vibrant*). Group 7, “attitude,” comprises adjectives indicating applicants’ affective perspectives, including evaluations and personal feelings (eg, *attractive*, *exciting*, and *incredible*). Findally, adjectives in group 8, “problem,” serve to problematize an issue (eg, *alarming*, *dire*, and *elusive*). Based on these groupings, the largest absolute increases were in importance (1342 wpm; 50%) and novelty (1790 wpm; 207%) (Figure 2).

## Discussion

Our analyses of adjectives indicate that the prevalence of hype language in abstracts of successful NIH grant proposals increased from 1985 to 2020. Our analyses also show that hype most often serves to promote the significance, novelty, scale, and rigor of the project; the utility of the expected outcomes; and the qualities of the investigators and research environment, as well as emphasizing the attitudes of the applicants and the gravity of the problem being addressed.

Although prior studies^10,11,13^ have identified similar, but less pronounced, trends in biomedical research articles, our study differs from these in design and scale, and in addressing the language of funding applications vs the language of research reports. Specifically, previous research has relied on searching samples of research articles for hyping items selected a priori by the researchers. In contrast, the present study was based on population-level data (ie, all records contained within the NIH archive), and terms were extracted from the data. This method allowed for the identification of a greater number of hype words, as well as reducing detection and sampling biases inherent in previous studies. To our knowledge, the list of hyping adjectives identified here (eTable in the Supplement) is more comprehensive than those previously available.

That we find hype language in grant applications is, in and of itself, not surprising. The genre is inherently promissory, requiring that applicants convince reviewers of the need for research, the means to conduct it, and its anticipated favorable contribution. In doing so, it appears that applicants increasingly describe their work in subjective terms and rely on appeals to emotion. For example, importance is described in absolute terms (eg, *imperative need*), novelty is sensationalized (*revolutionary innovation*), scale is amplified (*vast scale*), qualities are subjectified (*outstanding expertise*), and problems are dramatized (*dire need*).

This trend in language could be problematic. One concern is that hype might bias evaluation.^10,11^ It has been argued that hype may modulate readers’ interpretations of research reports, potentially to the detriment of patients, although this possibility remains to be convincingly demonstrated.^22,23,24^ Similarly, it may be that hype influences the evaluation of research funding applications. However, the present study provides no evidence in this regard because, although we have shown that successful applications use more hype, we did not have access to and therefore could not analyze unsuccessful applications.

Hype can undermine clarity. Although some hype terms can be considered standard, even required, many are gratuitous. Many represent buzzwords, which the Merriam-Webster dictionary defines as “important-sounding usually technical word[s] or phrase[s] often of little meaning used chiefly to impress laymen.”^25^ The little meaning that words such as *interdisciplinary* or *transformative* add to a text (much research occurs across disciplines and virtually all research seeks to effect change) could certainly be expressed in simpler terms. Hype can also lead to redundancy (eg, *strategic plan*, *new emerging technologies*, *novel innovations*, and *strong emphasis*). Such tautologies do little more than pad the word count and add to the processing burden on the reader, possibly obscuring important information (or the lack thereof).

Some pressures on grant applicants to use hype language may derive from the competitive nature of research funding and publishing, where increasing numbers of researchers are in competition with one another for academic appointments, tenure, and so forth.^26^ Also, pressures on researchers likely derive from the requirements of the funding bodies, which themselves have evolved over the years. Currently, the NIH specifically advises applicants that reviewers will pay particular attention to the likely overall “impact” of the proposed work and will specifically score proposals on the criteria of “significance” of the work, appropriateness of the “investigator(s),” “innovation,” the appropriateness of the “approach,” and the quality of the research “environment.”^27^ Under these circumstances, applicants may feel compelled to echo certain terms from agency guidance.

Furthermore, the increase in the use of hype in biomedical writing has not occurred in isolation; rather, it has taken place alongside broader societal changes. In 2020, 30% of abstracts of successful grants funded by the National Foundation of Science contained at least 1 term considered highly politicized (eg, *diversity*, *equity*, and *inclusion*), an increase from 2.9% in 1990.^28^ During the same period, language use in the more public domain of news media has shifted to now contain greater subjectivity, increased focus on first-person perspective, and more appeals to emotion.^29^ In addition, technological changes have enabled research on a scale that, compared with 36 years ago, can be described as “massive,” “enormous,” or “huge.”^30^ Furthermore, as the rate of technological change increases, it may be that novelty and innovation have become equated with progress and good research by many authors and readers.^31^

Finally, we point to natural processes of language change. An established mechanism of lexical change known as *semantic bleaching* involves a word being used with more exaggerated meanings (ie, hyperbole), until its overuse “bleaches” out the stronger meaning of the word.^32^ Arguably, the increased use of words such as “novel” and “critical” (occurring in 36% and 25% of all abstracts, respectively, in 2020) indicates progression toward meanings difficult to distinguish from “new” and “important.”

### Limitations

Our study has several limitations. First, we analyzed only public-facing abstracts for successful projects. Although the observed patterns may hold across the other sections of NIH funding applications, we are unable to demonstrate this. Second, although promotion can be realized through a range of lexical, grammatical, and rhetorical structures, our analysis was restricted to adjectives that have changed in frequency. Finally, despite good agreement between analysts, our categorization of adjectives was, necessarily, subjective in nature.

## Conclusions

Funding from the NIH is the *sine qua non* of health research in the United States, and, therefore, it has profound downstream effects on the entire research ecosystem. As such, the infusion of salesmanship at this stage of the research cascade, even if promotional language appears to be a minor force, may, over time and in consideration of the substantial NIH budget, influence the tone and substance of the entire research enterprise. The present study shows how the use of hype language in abstracts of successful NIH grant applications, as measured by the prevalence of promotional or value-laden adjectives, has increased substantially from 1985 to 2020. The increase in the use of hype in biomedical writing is not a phenomenon that occurs in isolation but likely reflects broader societal changes and the natural evolution of languages. In grant applications, the increase in the use of hype may also be in response to competition for rewards and instructions from funding agencies. To the extent that spin, in general, and hype, in particular, may influence the evaluation and writing of funding applications, these findings should serve to sensitize applicants, reviewers, and funding agencies to the increasing prevalence of value-laden language. Future work should explore how language used in funding opportunity announcements is replicated in applications and subsequent publications.

## Notes

Supplement. **eTable.** Frequency in and Change of 139 Hype Adjectives Between 1985 and 2020 **eFigure.** Yearly Frequencies for All Hype Adjectives From 1985 to 2020 in Words per Million (wpm)
