# Supplementary material for: Trends in the Use of Promotional Language (Hype) in Abstracts of Successful National Institutes of Health Grant Applications, 1985-2020
Source: JAMA Netw Open. 2022 Aug 25;5(8):e2228676. doi: 10.1001/jamanetworkopen.2022.28676 (PMC9412227; doi:10.1001/jamanetworkopen.2022.28676)
Supplement: Supplement. — eTable. Frequency in and Change of 139 Hype Adjectives Between 1985 and 2020 eFigure. Yearly Frequencies for All Hype Adjectives From 1985 to 2020 in Words per Million (wpm) [file jamanetwopen-e2228676-s001.pdf]

Supplementary Online Content

Millar N, Batalo B, Budgell B. Trends in the use of promotional language (hype) in abstracts of successful National Institutes of Health grant applications, 1985-2020. *JAMA Netw Open.* 2022;5(8):e2228676.
doi:10.1001/jamanetworkopen.2022.28676

eTable. Frequency in and Change of 139 Hype Adjectives Between 1985 and 2020
eFigure. Yearly Frequencies for All Hype Adjectives From 1985 to 2020 in Words per Million (wpm)

This supplementary material has been provided by the authors to give readers additional information about their work.

© 2022 Millar N et al. *JAMA Network Open.*

eTable. Frequency in and Change of 139 Hype Adjectives Between 1985 and 2020

Adjective	Adjective frequency		Overall change	
	1985	2020	absolute	relative
novel	1056	12809	+1054 wpm	+717%
critical	1147	7623	+555 wpm	+348%
key	742	6013	+461 wpm	+446%
innovative	137	4368	+391 wpm	+2048%
scientific	581	4422	+334 wpm	+413%
effective	1674	5836	+314 wpm	+135%
successful	743	3966	+269 wpm	+260%
diverse	388	3333	+259 wpm	+479%
significant	1974	5600	+250 wpm	+91%
advanced	290	2383	+183 wpm	+454%
robust	35	1948	+178 wpm	+3652%
relevant	1246	3694	+173 wpm	+100%
strong	441	2451	+168 wpm	+275%
unique	1750	4199	+150 wpm	+62%
comprehensive	626	2330	+131 wpm	+151%
broad	595	1900	+95 wpm	+115%
essential	1411	3081	+93 wpm	+47%
rigorous	105	1092	+88 wpm	+601%
promising	265	1318	+87 wpm	+235%
interdisciplinary	231	1188	+79 wpm	+247%
urgent	16	816	+74 wpm	+3332%
quality	149	989	+72 wpm	+347%
first	3175	5440	+68 wpm	+15%
unmet	6	729	+68 wpm	+8039%
outstanding	55	764	+64 wpm	+837%
efficient	474	1385	+64 wpm	+97%
substantial	321	1156	+64 wpm	+143%
multidisciplinary	478	1341	+59 wpm	+89%
crucial	347	1105	+55 wpm	+115%
strategic	37	625	+53 wpm	+1038%
unprecedented	31	606	+53 wpm	+1219%
fundamental	1072	2130	+51 wpm	+34%
timely	64	628	+50 wpm	+561%
scalable[a]	0	476	+45 wpm	+13029%
largest	91	610	+45 wpm	+352%
powerful	315	940	+44 wpm	+101%
extensive	763	1597	+44 wpm	+41%
top	25	490	+42 wpm	+1221%
international	124	631	+42 wpm	+243%
exciting	62	536	+42 wpm	+483%
devastating	30	466	+40 wpm	+946%
senior	64	508	+39 wpm	+435%

© 2022 Millar N et al. *JAMA Network Open.*

ideal	179	661	+37 wpm	+149%
sustainable	1	377	+35 wpm	+25157%
accessible	194	660	+35 wpm	+129%
rich	260	750	+34 wpm	+94%
accurate	434	995	+33 wpm	+55%
exceptional	17	372	+33 wpm	+1372%
emerging	13	335	+30 wpm	+1636%
vast	63	404	+29 wpm	+332%
intellectual	95	448	+29 wpm	+218%
productive	132	500	+29 wpm	+155%
meaningful	114	450	+26 wpm	+166%
compelling	35	326	+26 wpm	+528%
transformative[a]	0	274	+26 wpm	+8190%
efficacious	78	386	+25 wpm	+233%
foundational	0	263	+25 wpm	+6226%
synergistic	123	443	+24 wpm	+143%
transdisciplinary[a]	0	255	+24 wpm	+7616%
ready	53	333	+24 wpm	+323%
easy	67	352	+24 wpm	+254%
qualified	54	331	+24 wpm	+313%
durable	6	253	+23 wpm	+2725%
actionable[a]	0	242	+23 wpm	+16114%
elusive	25	279	+23 wpm	+652%
impactful[a]	0	238	+22 wpm	+6465%
deeper	47	301	+22 wpm	+332%
vital	222	543	+20 wpm	+65%
tremendous	28	254	+20 wpm	+511%
greatest	103	358	+19 wpm	+134%
latest	33	243	+18 wpm	+397%
enormous	42	255	+18 wpm	+309%
sophisticated	131	377	+17 wpm	+94%
pivotal	62	266	+16 wpm	+189%
imperative	32	212	+15 wpm	+347%
reproducible	114	318	+14 wpm	+88%
generalizable	16	166	+13 wpm	+598%
experienced	36	194	+13 wpm	+263%
longstanding	14	159	+13 wpm	+665%
unparalleled	6	142	+12 wpm	+1486%
user-friendly	15	154	+12 wpm	+591%
attractive	88	259	+12 wpm	+98%
talented	5	129	+11 wpm	+1629%
invaluable	43	185	+11 wpm	+190%
remarkable	80	229	+10 wpm	+93%
safer	49	183	+10 wpm	+152%
skilled	31	152	+10 wpm	+231%
massive	66	204	+10 wpm	+108%

© 2022 Millar N et al. *JAMA Network Open.*

seamless	1	100	+9 wpm	+6600%
unanswered	46	161	+9 wpm	+136%
notable	19	110	+8 wpm	+291%
vibrant[a]	0	77	+7 wpm	+903%
huge	6	85	+7 wpm	+849%
intriguing	4	81	+7 wpm	+145%
alarming	35	127	+7 wpm	+1257%
creative	9	84	+7 wpm	+530%
motivated	24	104	+6 wpm	+192%
paramount	16	92	+6 wpm	+287%
renowned[a]	0	68	+6 wpm	+1776%
surprising	30	112	+6 wpm	+151%
indispensable	10	82	+6 wpm	+453%
myriad	4	72	+6 wpm	+1105%
tailored	1	64	+6 wpm	+4186%
ample	16	85	+6 wpm	+257%
fastest	8	73	+6 wpm	+517%
cohesive	19	89	+6 wpm	+216%
premier	1	61	+6 wpm	+3986%
immense	8	71	+6 wpm	+500%
ambitious	6	62	+5 wpm	+592%
overwhelming	15	75	+5 wpm	+236%
dismal	2	54	+5 wpm	+1707%
revolutionary	3	55	+5 wpm	+1129%
confident	10	65	+5 wpm	+339%
dedicated	2	52	+5 wpm	+1643%
biggest	3	50	+4 wpm	+1017%
dire	1	46	+4 wpm	+2979%
nuanced[a]	0	43	+4 wpm	+2779%
intuitive	4	48	+4 wpm	+704%
tangible	3	42	+4 wpm	+838%
incredible[a]	0	36	+3 wpm	+894%
daunting[a]	0	35	+3 wpm	+1115%
collegial[a]	0	32	+3 wpm	+782%
interprofessional[a]	0	32	+3 wpm	+868%
deployable[a]	0	32	+3 wpm	+1150%
expansive	1	32	+3 wpm	+2043%
groundbreaking[a]	0	25	+2 wpm	+655%
prestigious[a]	0	24	+2 wpm	+562%
stellar[a]	0	24	+2 wpm	+626%
stark[a]	0	22	+2 wpm	+122%
desperate[a]	0	22	+2 wpm	+565%
careful	188	100	-17 wpm	-64%
systematic	489	520	-19 wpm	-28%
interesting	217	87	-22 wpm	-73%
immediate	559	579	-24 wpm	-30%

© 2022 Millar N et al. *JAMA Network Open.*

ultimate	604	499	-37 wpm	-44%
considerable	538	392	-38 wpm	-51%
detailed	1603	1086	-121 wpm	-54%
important	5569	6695	-147 wpm	-19%
major	5619	5557	-261 wpm	-33%

Abbreviations**:** wpm = words per million - number of words in 1985 = 7,185,061; number of words in 2020 = 10,662,335
[a]Adjective was absent in 1985 – percentage change calculated based on first year of occurrence

© 2022 Millar N et al. *JAMA Network Open.*

eFigure. Yearly Frequencies for All Hype Adjectives From 1985 to 2020 in Words per Million (wpm)

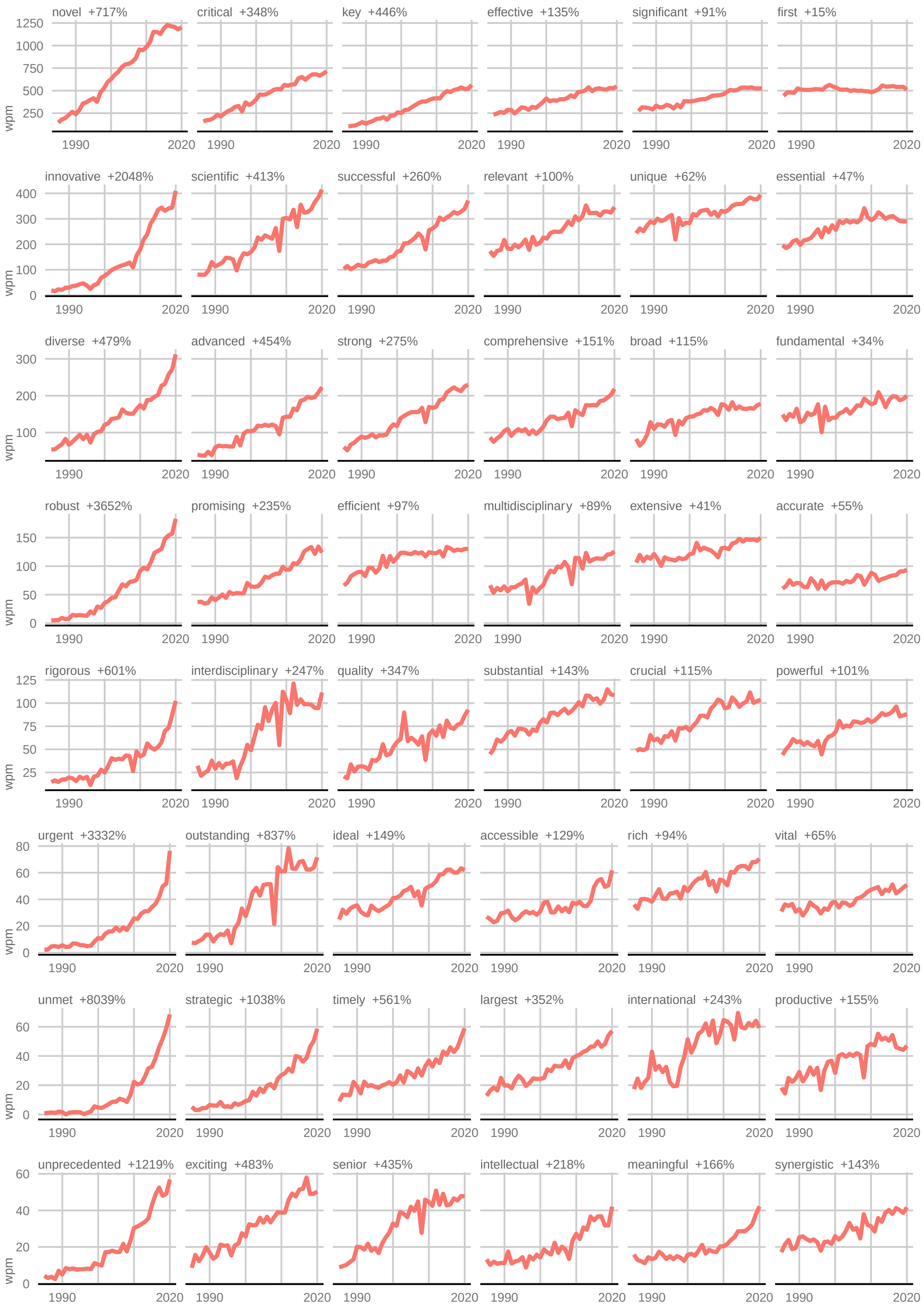

© 2022 Millar N et al. *JAMA Network Open.*

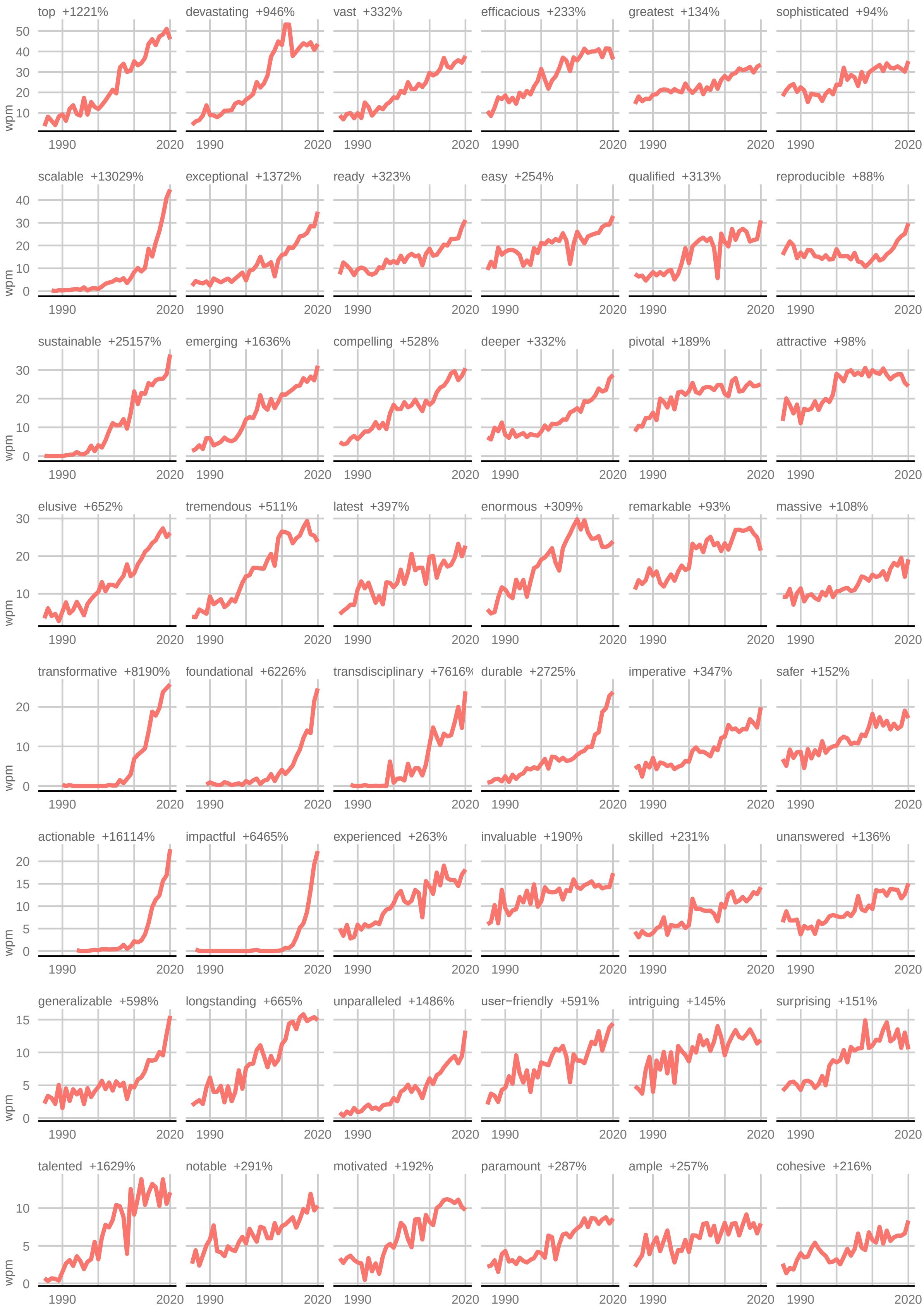

© 2022 Millar N et al. *JAMA Network Open.*

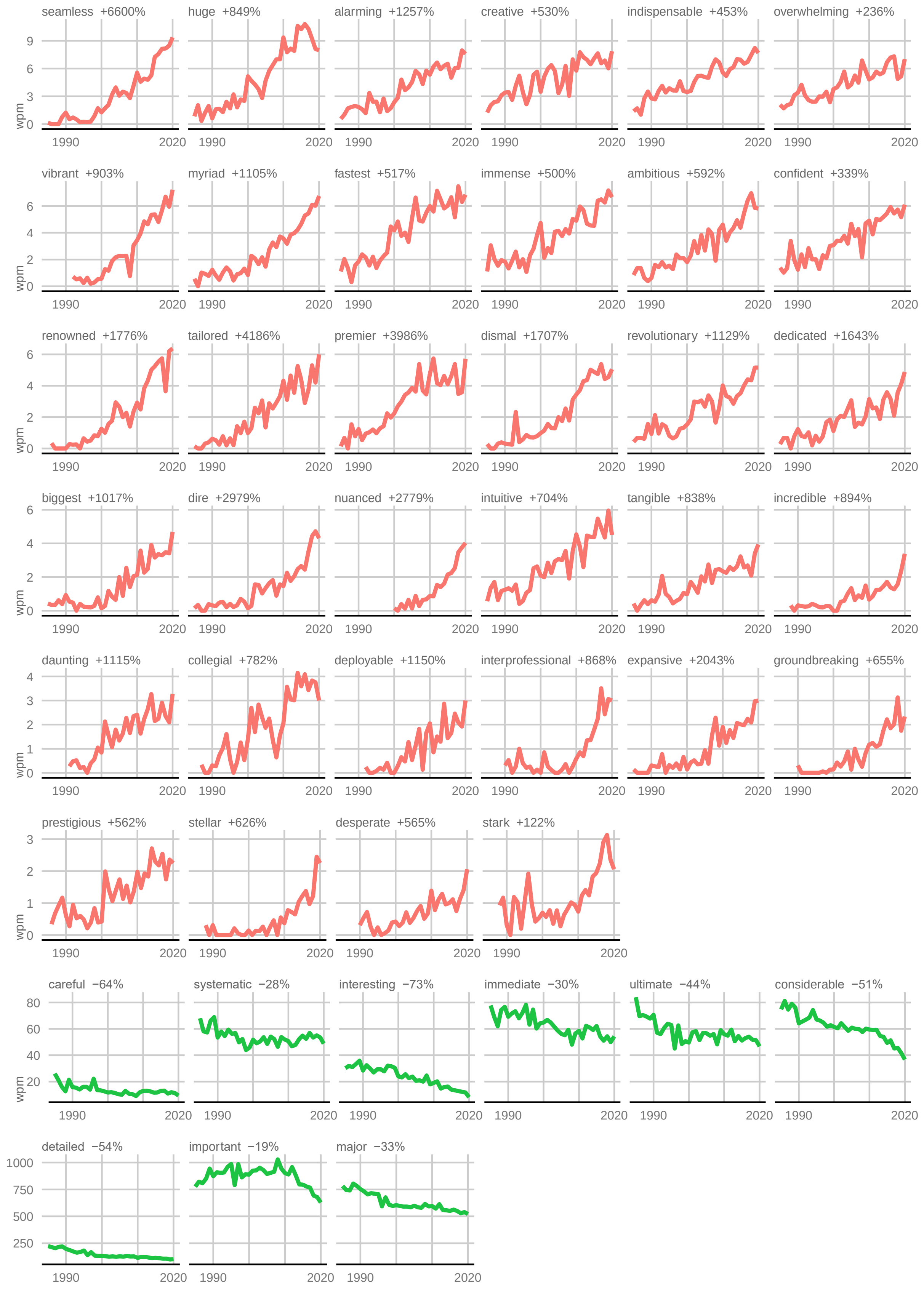

Abbreviations: wpm = words per million

© 2022 Millar N et al. *JAMA Network Open.*
